# Radiation damage in macromolecular crystallography: what is it and why should we care?

**DOI:** 10.1107/S0907444910008656

**Published:** 2010-03-24

**Authors:** Elspeth F. Garman

**Affiliations:** a Laboratory of Molecular Biophysics, Department of Biochemistry, University of Oxford, South Parks Road, Oxford OX1 3QU, England

**Keywords:** radiation damage, cryocrystallography, room-temperature macromolecular crystallography, dose

## Abstract

Radiation damage inflicted during diffraction data collection in macromolecular crystallography has re-emerged in the last decade as a major experimental and computational challenge, as even for crystals held at 100 K it can result in severe data-quality degradation and the appearance in solved structures of artefacts which affect biological interpretations. Here, the observable symptoms and basic physical processes involved in radiation damage are described and the concept of absorbed dose as the basic metric against which to monitor the experimentally observed changes is outlined. Investigations into radiation damage in macromolecular crystallography are ongoing and the number of studies is rapidly increasing. The current literature on the subject is compiled as a resource for the interested researcher.

## Abstract 2

The basic causes of the radiation damage inflicted on macromolecular crystals during diffraction experiments are summarized, as well as the current state of research which attempts to understand and to mitigate it.

## 1. Introduction

The advent of highly intense wiggler and undulator beamlines fed from synchrotron sources has reintroduced the age-old problem of X-ray radiation damage in macromolecular crystallography (MX) even for crystals held at cryogenic temperatures (100 K). Unfortunately, such damage to macromolecular crystalline samples during the experiment is a problem that is inherent in using ionizing radiation to obtain diffraction patterns and has presented a challenge to MX since the beginning of the field. For room-temperature (RT) data collections, it often necessitates the use of many crystals to assemble a complete data set, because the crystalline order of the sample is damaged and decreases during the experiment and thus the diffracted intensity fades. The root cause of this damage is the energy lost by the beam in the crystal owing to either the total absorption or the inelastic scattering of a proportion of the X-rays as they pass through the crystal. The measure of this energy loss is the ‘dose’ measured per mass of the sample, given in SI units of grays (Gy; 1 Gy = 1 J kg^−1^). Dose may also be quoted in terms of the non-SI unit rad (radiation absorbed dose; 1 rad = 10 mGy). In MX, dose measurements are generally of the order of a million grays (1 MGy or 100 Mrad).

The earliest investigation of radiation damage at RT in MX was carried out nearly 50 years ago by Blake & Phillips (1962 ▶) on a sealed-tube (copper) X-ray source. By making seven sets of successive measurements, they monitored the decay in the diffraction intensity of a particular set of reflections from crystals of sperm-whale myoglobin over a period of 300 h. They concluded that the damage was proportional to the irradiation time, which they assumed was linearly proportional to the absorbed dose. They deduced that a single 8 keV X-ray photon disrupts around 70 protein molecules and disorders a further 90 protein molecules for doses up to about 20 Mrad (0.2 MGy) absorbed after 100 h of X-ray exposure. The observed form of the decay with dose could be described by an exponential function representing a first-order process, 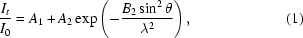
where *I*_*t*_ corresponds to the measured intensity at a particular time, *I*_0_ is the initial intensity, *B* is a measure of disorder, θ is the angle of diffraction and λ is the incident X-ray wavelength. According to their model, after any irradiation the crystal consists of three components: (i) an undamaged fraction (*A*_1_) which is entirely responsible for the remaining diffraction at high angles, (ii) a highly disordered fraction (*A*_2_) which only con­tributes to the diffraction at low angles and (iii) a thoroughly disorganized or amorphous part [1 − (*A*_1_ + *A*_2_) = *A*_3_] which no longer contributes to the single-crystal diffraction at all. From their plot of *A*_1_, *A*_2_ and *A*_3_ against time derived from the seven successive sets of measurements and using their dose estimates it can be deduced that half of the crystal volume became amorphous after a dose of 0.59 MGy.

Blake & Phillips (1962 ▶) also suggested that the protein molecules suffered specific structural damage. This conclusion was reached without knowledge of either the sequence or the three-dimensional structure of the protein, and the postulate was only confirmed many years later when radiation damage to disulfide bridges was noted in electron-density difference maps calculated from data collected from des-pentapeptide insulin crystals (Helliwell, 1988 ▶) as well as the opening of aromatic side chains in maps of ribonuclease (unpublished results from Burley and coworkers referred to in Helliwell, 1988 ▶). As early as 1958, it was postulated that covalent bonds in proteins provided a migratory route for ionizing energy from absorbed incident radiation to break weaker bonds (Augenstein, 1958 ▶). Breakage of disulfide bonds had been reported following the irradiation of solutions of the proteins trypsinogen and chymotryspinogen by 186 keV electrons (produced by the decay of the radionuclide ^35^S; Pechère *et al.*, 1958 ▶). The presence of sulfur radicals and the subsequent formation of —SH groups was confirmed by ESR measurements (Gordy & Shields, 1958 ▶).

Following the work of Blake & Phillips (1962 ▶), various researchers (Hendrickson *et al.*, 1973 ▶; Hendrickson, 1976 ▶; Fletterick *et al.*, 1976 ▶) investigated the radiation-damage problem in protein crystals both theoretically and experimentally at RT and made modifications to the initial model presented above. A detailed description of these developments can be found in the literature (Southworth-Davies *et al.*, 2007 ▶) and will not be repeated here. The resulting working model for RT damage which fitted all the available data was that there appeared to be no direct pathway between states *A*_1_ and *A*_3_ and thus the rate constant for transition from un­damaged to amorphous was zero. Additionally, it was found necessary to include an intermediate dose-dependent stage labelled *A*_1_′ between the undamaged and the damaged stages as shown in (2). This state conformationally resembled the undamaged state and thus still contributed to diffraction at all angles (Sygusch & Allaire, 1988 ▶),
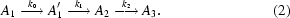
Up until the 1990s, MX data were almost exclusively collected at RT, where the recommended practice was to monitor the intensity *I*_0_ of a strong reflection as the experiment proceeded and to discard the crystal once the intensity had dropped to 0.85*I*_0_, or at the very worst 0.70*I*_0_ if the particular crystals were in very short supply (Blundell & Johnson, 1976 ▶).

Much earlier, improved resolution of diffraction had been observed for crystals held at 246 K (King, 1958 ▶), although at the time this was not understood in terms of reduced radiation damage. Systematic measurements comparing the decay of two particular reflections for crystals held at 198 and 298 K (Haas & Rossmann, 1970 ▶) and efforts to import small-molecule crystallography cooling techniques into MX (Hope, 1988 ▶) showed that this would be an effective radiation-damage mitigation strategy. By irradiating the crystal while holding it at a reduced temperature, its lifetime should be significantly improved, since many of the radical species produced by the energy loss of the beam would diffuse much more slowly or not at all and would thus not further interact, so reducing the collateral damage.

The cryocooling technique blossomed and was made technically more accessible for routine use in MX because of two pivotal developments: the loop-mounting method (Teng, 1990 ▶), in which the protein crystal is held by surface tension in a film of liquid ‘cryo-buffer’ across a small-diameter (1 mm down to 0.1 mm) nylon or fibre loop, and the availability of a reliable open-flow unpressurized cryostat with flexible stainless-steel hosing (Cosier & Glazer, 1986 ▶) to supply a stream of cooled gaseous nitrogen at a stable temperature of around 100 K with which to surround the sample during data collection. Initially, problems with the technique included ice formation within and outside the crystal and an increase in mosaic spread, particularly when cryocooling protocols were not optimized. Methods for improving the data quality obtainable were soon developed (Rodgers, 1997 ▶; Garman & Schneider, 1997 ▶; Garman, 1999 ▶; Pflugrath, 2004 ▶; Garman & Owen, 2006 ▶) and there was widespread adoption of the technique. In fact it has been estimated that over 90% of all protein structures are now determined at cryo-temperatures (Garman, 2009 ▶).

The advantages of cryocooling for MX are a reduction in the rate of radiation damage; the use of a mounting technique (the loop) that is usually more gentle than the capillary method historically used for RT collection; the fact that higher resolution data can more easily be obtained because the crystal order is preserved for longer; a lower background in the diffraction experiment as it is not necessary to enclose the crystal in a glass, quartz or plastic tube to prevent dehydration; that fewer crystals (and thus a lower quantity of protein) are required for a project; that crystals can be shipped ahead of time to the synchrotron (more or less) safely; and that crystals can be flash-cooled when in peak condition for future use before they start to degrade in the crystallization drop.

These positive aspects of cryocooling commonly outweigh the disadvantages. The latter include the requirement for expensive cryostat cooling equipment, a frequent increase in crystal mosaic spread (but not necessarily if the cryoprotection concentration and crystal handling are carefully optimized), the need to invest time for optimization of cryo-buffers and cooling protocols, and the fact that there are as yet no protocols that guarantee success, although progress is being made in this direction (see, for example, Alcorn & Juers, 2010 ▶).

The improvement in dose tolerance for a crystal held at 100 K compared with a crystal irradiated at RT has been estimated to be approximately a factor of 70 on average (Nave & Garman, 2005 ▶). Thus, cryocooling is clearly a highly effective mitigation strategy. However, radiation damage is now routinely observed at synchrotrons in cryocooled crystals and the experimenter would be wise to be aware of the artefacts that can be produced. Below, the symptoms of radiation damage at cryotemperatures and the basic physical processes involved are described, the reasons why the crystallographer should care about this issue are addressed, and our current knowledge, as reflected in the published literature, is collated. The interested reader is also referred to Garman & Owen (2006 ▶) and Ravelli & Garman (2006 ▶), and to a recent article entitled *A beginner’s guide to radiation damage* (Holton, 2009 ▶).

## 2. What are the symptoms of radiation damage at cryotemperatures?

**Figure 1:**
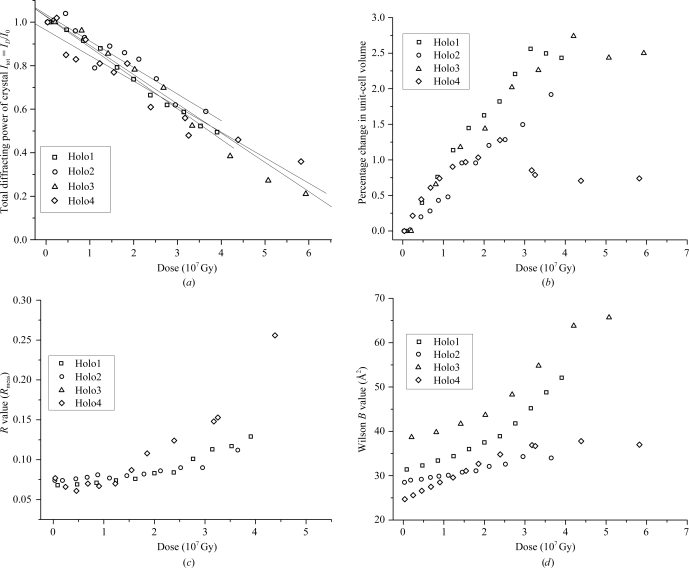
Global radiation-damage indicators as a function of dose for four holoferritin crystals (Owen *et al.*, 2006 ▶). (*a*) Mean *I*/mean *I*_0_, (*b*) unit-cell volume, (*c*) *R* value and (*d*) Wilson *B* value.

**Figure 2:**
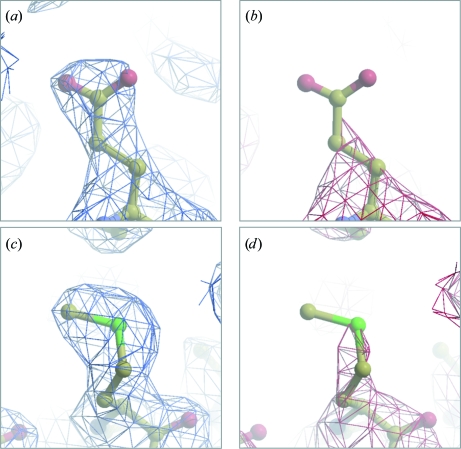
Specific structural damage inflicted on a cryocooled crystal of apoferritin during sequential data sets collected on beamline ID14-4 at ESRF. (*a*) 2*F*_o_ − *F*_c_ map of Glu63 contoured at 0.2 e Å^−3^ after a dose of 2.5 MGy and (*b*) after 50 MGy. (*c*) 2*F*_o_ − *F*_c_ map of Met96 contoured at 0.2 e Å^−3^ after a dose of 2.5 MGy and (*d*) after 50 MGy, showing loss of electron density around the disordered atoms (Garman & Owen, 2006 ▶).

**Figure 3:**
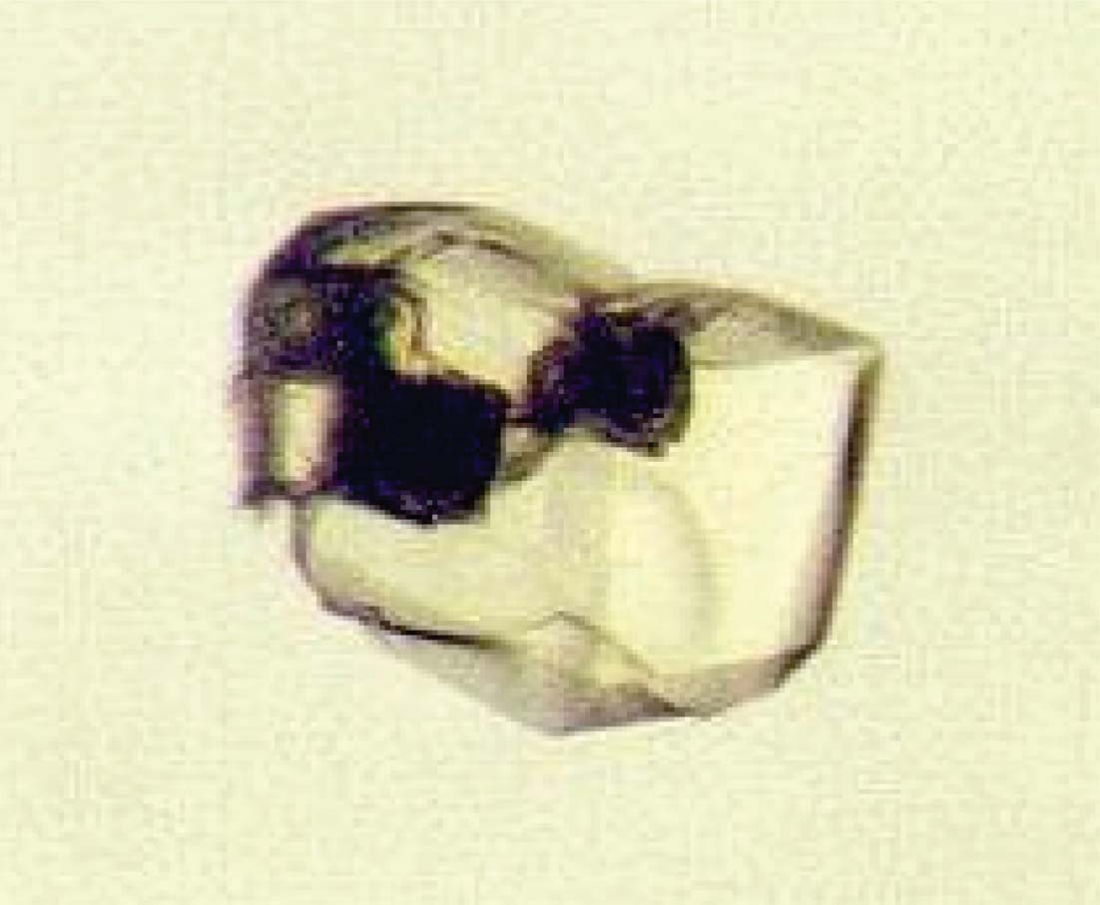
Photograph of a 400 µm neuraminidase crystal (subtype N9 from avian influenza isolated from a noddy tern), space group *I*432, that has been irradiated on ID14-4 at the ESRF at 100 K and then allowed to warm up to RT. The three black marks are from the 100 × 100 µm beam; the discolouration is an indication of radiation damage.

Systematic studies of this phenomenon have identified two separate indicators of damage as a function of dose: global (Fig. 1 ▶) and specific (Fig. 2 ▶) damage. The former results in a loss of the measured reflection intensities (particularly at high resolution), expansion of the unit-cell volume, increasing values of the measure of the internal consistency of the data which quantifies the difference between reflection intensities that should ideally be the same (*R*_meas_), an increase in both the scaling *B* factors for the data and the atomic *B* values of the refined structure, rotation of the molecule within the unit cell and often (but not always) an increase in mosaicity. Visible differences in the samples as the experiment proceeds, including colour changes, are also observed. On warming of the sample following irradiation, bubbles of gas, now proposed to be hydrogen (Meents *et al.*, 2009 ▶, 2010 ▶) and perhaps some CO_2_, are emitted and discolouration of the sample is common (see Fig. 3 ▶).

Various metrics have been suggested and used for monitoring global damage, among which are the following.

**Figure 4:**
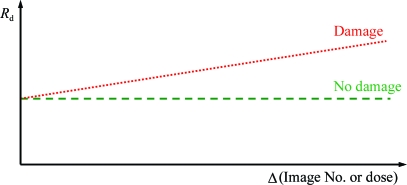
An idealized plot of *R*_d_, the pairwise *R* factor between identical and symmetry-related reflections occurring on different diffraction images, plotted against the difference in dose, Δ*D*, between the images on which the reflections were collected (Diederichs, 2006 ▶). The plot is a straight line parallel to the *x* axis if there is no damage, but rises linearly in the presence of damage.

**Figure 5:**
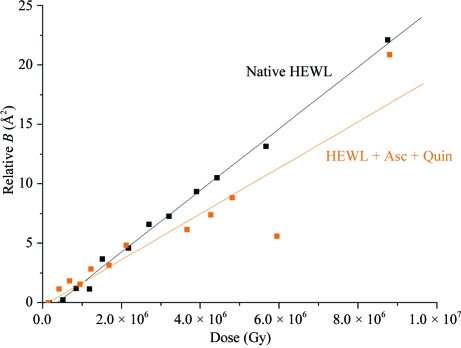
A plot of *B*_rel_ (one value per data set collected on ID14-4 at the ESRF) against dose for two HEWL crystals, one native and the other cocrystallized with the scavengers ascorbate (Asc) and 1,4-benzoquinone (Quin). The solid lines represent linear fits to the data: the increase in *B*_rel_ is only marginally slower with dose for the scavenger cocrystals, showing (when combined with an analysis of the resulting electron-density maps) that this particular combination is not effective in reducing the rate of damage (Southworth-Davies, 2008 ▶).

(i) *I*_*D*_/*I*_1_, where *I*_*D*_ is the summed mean intensity (*I*_mean_) of a complete data set (or equivalent sections of data) after a dose *D* and *I*_1_ is the mean intensity of the first data set. Note that using *I*_*D*_/σ_*D*_ (where σ_*D*_ is the standard deviation of the signal, *i.e.* the ‘noise’) normalized to the intensity *I*_1_/σ_1_ of the first data set is not a robust metric since the noise σ_*D*_ increases with dose and thus *I*_*D*_/σ_*D*_ reduces by an amount that more than represents the true loss of diffracting power.
(ii) *R*_d_, the pairwise *R* factor between identical and symmetry-related reflections occurring on different diffraction images, plotted against the difference in dose, Δ*D*, between the images on which the reflections were collected (Diederichs, 2006 ▶). The plot of *R*_d_ against Δ*D* is a straight line parallel to the *x* axis if there is no damage, but rises linearly in the presence of damage (see Fig. 4 ▶). This plot can be used to correct the intensity values of the reflections back to their ‘zero-dose’ values to improve the data quality (Diederichs *et al.*, 2003 ▶).
(iii) The isotropic *B* factor (*B*_rel_) has been found to be a robust measure of radiation damage at 100 K and to be linearly dependent on it (Kmetko *et al.*, 2006 ▶). An example of *B*_rel_ plotted against dose is given in Fig. 5 ▶. The relative *B* factors can be interpreted as proportional to the change in the mean-squared atomic displacements. A coefficient of sensitivity to absorbed dose, *S*_AD_, was also defined, *S*_AD_ = Δ*B*_rel_/Δ*D*8π^2^, where Δ*B*_rel_/8π^2^ is the change in relative isotropic *B* factor and Δ*D* is the change in dose as above, *i.e. S*_AD_ is the slope of the line in a graph such as that shown in Fig. 5 ▶. This metric relates the increase in mean-squared atomic displacements to the dose and it has been postulated that it is similar within quite a narrow range of values for most protein crystals (Kmetko *et al.*, 2006 ▶).
(iv) The volume of the unit cell increases more or less linearly with dose and was originally thought to be a possible metric for judging the extent of radiation damage; however, systematic work (Murray & Garman, 2002 ▶; Ravelli *et al.*, 2002 ▶) has shown that it is not a reliable indicator since crystals of the same size and type expand at different rates with increasing absorbed dose.
(v) Although mosaicity commonly increases with dose, it is  not a reliable metric for quantization of radiation damage, since it does not behave in a reproducible or predictable manner.

Of more direct relevance to the biological interpretation of structures than the global indicators detailed above is the fact that specific structural damage to particular covalent bonds is observed to occur in a reproducible order in many proteins (Weik *et al.*, 2000 ▶; Burmeister, 2000 ▶; Ravelli & McSweeney, 2000 ▶): first disulfide bridges elongate and then break (Weik *et al.*, 2002 ▶), then glutamates and aspartates are decarboxylated, tyrosine residues lose their hydroxyl group and subsequently the carbon–sulfur bonds in methionines are cleaved. Such damage is illustrated in Fig. 2 ▶, which shows damage to glutamate and methionine residues in a cryocooled crystal of apoferritin during sequential data sets collected on beamline ID14-4 at the ESRF. Covalent bonds to heavier atoms such as C—Br, C—I and S—Hg are also ruptured (see, for example, Ramagopal *et al.*, 2005 ▶).

Clearly, it is not feasible to monitor the specific structural damage during the experiment, since the refined structures are required. However, it is known that this damage often occurs well before there is any obvious degradation of the diffraction pattern.

The global effects of radiation damage at 100 K are thought to be independent of dose rate up to the flux densities currently used (10^15^ photons s^−1^ mm^−2^; Sliz *et al.*, 2003 ▶). Another study concurred with this finding but, following an analysis of electron-density difference maps, indicated that there might be a second-order dose-rate effect since specific damage was slightly more severe at higher dose rates (Leiros *et al.*, 2006 ▶). Conversely, however, Owen *et al.* (2006 ▶) reported a small (10%) reduction in *D*_1/2_ (the dose required to halve the original diffraction intensity) for a dose-rate increase from 4 × 10^3^ to 40 × 10^3^ Gy s^−1^ at flux densities of 4 × 10^12^ and 4 × 10^13^ photons s^−1^ mm^−2^, respectively.

**Figure 6:**
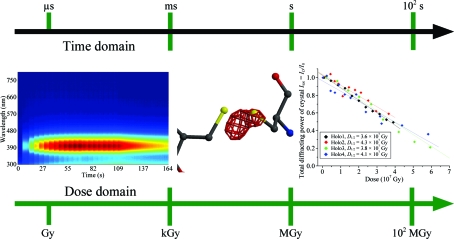
Illustration of radiation damage over a wide range of time scales and dose. Left, UV–vis absorption spectrum (blue, lowest; red, highest) of a cryocooled solution of cysteine, showing an intense peak at 400 nm corresponding to disulfide-anion radical production. The vertical bands arise from 1 s X-ray irradiations followed by 5–8 s of beam off, during which the 400 nm peak decays away (Southworth-Davies & Garman, 2007 ▶). Centre, *F*_o_ − *F*_c_ difference density map (contoured at −2.5σ) of the Cys76–Cys94 bond in a HEWL structure calculated using the sixth data set in a sequential collection from one crystal (Murray & Garman, 2002 ▶). The bond is broken and the S atoms are delocalized. Right, decay of the normalized diffraction intensity of sequential data sets collected from four different holoferritin crystals (Owen *et al.*, 2006 ▶). Figure modified from Owen, Pearson *et al.* (2009 ▶).

The manifestations of radiation damage in the diffraction experiment can now be monitored over a range of time scales and doses (illustrated in Fig. 6 ▶). For instance, the formation of the disulfide-anion radical, 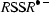, can be observed in real time using UV/UV–vis microspectrophotometry after a few tens of milliseconds of X-ray irradiation as a 400 nm absorption peak, and solvated electrons have a maximal absorbance at 550–600 nm (McGeehan *et al.*, 2009 ▶). This specific structural damage is often apparent in electron-density maps calculated using the structure factors of a data set that took around 30 s to collect and the resulting structure represents a time and space average over the 30 s of irradiation and over all the molecules in the crystal (Fig. 6 ▶). Metal centres are also reduced very swiftly by the X-ray beam and increasingly this can be monitored on-line during the X-ray experiment (see, for example, Hough *et al.*, 2008 ▶). The global intensity loss owing to radiation damage is clearly evident following the collection of several data sets in succession from the same crystal when the summed intensity for each data set is plotted normalized to the intensity of the first data set (Fig. 6 ▶, right).

## 3. What is it?

**Figure 7:**
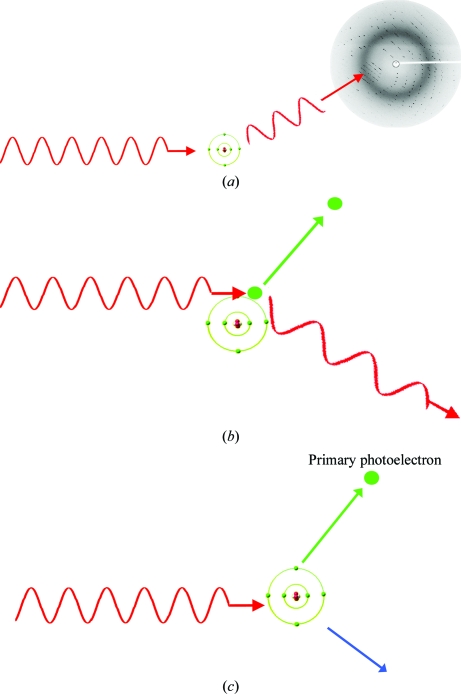
Primary X-ray interaction processes with the atoms of the crystal and solvent. (*a*) Elastic (Thomson, coherent) scattering. The waves are phase-shifted by 180° on scattering and add vectorially to give the diffraction pattern. (*b*) Compton (incoherent) scattering. The X-ray transfers some energy to an atomic electron and thus has lower energy (higher wavelength) after the interaction. Energy is lost in the crystal, contributing to the absorbed dose. (*c*) Photoelectric absorption. The X-ray transfers all its energy to an atomic electron, which is then ejected and can give rise to the ionization of up to 500 other atoms. The excited atom can then emit a characteristic X-ray or an Auger electron to return to its ground state.

Radiation damage to the sample is a result of it absorbing photons from the beam by either the photoelectric effect (total absorption of the photon and ejection of an inner shell electron) or Compton scattering (inelastic scattering of the photon, which then escapes following a varying amount of energy loss to an atomic electron, which can also be ejected). At the incident energies used for MX, the former effect has a much higher cross-section and dominates the absorption, accounting for over 90% of the energy deposited by the beam. Each photoelectron has enough energy to subsequently induce up to ∼500 further ionization events, which in turn can result in the formation of radical species in the crystal. In protein crystals, the presence of anything between 20% and 80% solvent means that the radiolysis of water and other components of the solvent is an important contributor to the creation of these species. Some of the energy deposited by the beam during these processes is converted into heat and induces a temperature rise in the sample. The diffracted photons are scattered elastically and thus do not contribute to the damage. These processes are illustrated diagrammatically in Figs. 7 ▶(*a*), 7 ▶(*b*) and 7 ▶(*c*). It is worth noting that for a 100 µm thick protein crystal only 2% of the incident photons of a 12.4 keV (1 Å) X-ray beam will interact in any way with it [∼1.7% (*i.e.* 84% of interacting photons) by the photoelectric effect and ∼0.15% (8%) by the Compton effect, with only ∼0.15% (8%) actually diffracting].

The usage of the terms ‘primary’, ‘secondary’ and ‘tertiary’ damage has become somewhat inconsistent in the literature and is largely a matter of semantics, but the definitions that will be adopted here are as follows.

(i) Primary damage is the ionization of an atom owing to photoelectric absorption or Compton scattering. The primary photoelectron has a mean track length of a few micrometres (for 12 keV photons; O’Neill *et al.*, 2002 ▶).
(ii) Secondary damage is that arising from the formation of up to 500 low-energy secondary electrons per primary absorption event, which are able to diffuse and induce further ionization and excitation events (*e.g.* electronic and vibrational). The secondary electrons gradually become thermalized (that is, they have the distribution of energies expected at the equilibrium temperature of the sample) and chemical reactions between the radiation-induced moieties and the crystal components then become important.
(iii) Tertiary damage is defined as the effect on the crystal lattice and other mechanical consequences of the energy deposition in the crystal.

Damage can also be classified as direct, if the primary absorption event occurs at an atom in the protein molecule, or indirect, if the radiation is absorbed by the surrounding solvent and the reactive species formed subsequently interact with the protein. Energy deposition in the water in and around the crystal results in a cascade of reactions as shown below (Ward, 1988 ▶), giving hydroxyl radicals, hydrated electrons and H atoms, the relative amounts of which depend on the temperature, pH and other factors,
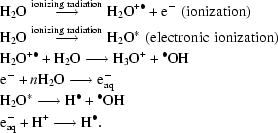
As described in §[Sec sec2]2 above, the knock-on effects of the energy absorbed by the crystal manifest themselves as both a reduction of crystalline order (global damage) and specific structural damage, and over the last 10 years MX researchers have sought to identify mechanisms that explain these observations.

At RT the products have thermal energy and can diffuse through the crystal, causing more secondary damage as they go. However, at cryotemperatures below 110 K nearly all the radical species, including 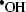 radicals (Mike Sevilla, private communication), are immobilized, with the notable exception of electrons. These can quantum-mechanically tunnel along the amino-acid backbone and have been shown by ESR measurements to be mobile at 77 K (Jones *et al.*, 1987 ▶). They migrate and seek out the most electron-affinic sites in the protein which, if there are no bound metals, are the disulfide bonds. This phenomenon accounts for the ‘pecking order’ of amino acids susceptible to specific structural damage. This mechanism also explains why the observed damage does not occur in the order of the largest to smallest X-ray absorption cross-sections of the atoms, as would be expected if there were no mobile species. Away from absorption edges, X-ray absorption cross-sections rise swiftly with the atomic number of an atom, so if the specific structural damage arose from primary processes alone the C—S bond in methionine should be the second most susceptible bond (after the disulfide bond).

The reason for the global damage to crystalline order observed in MX was until recently thought to be the consequence of direct damage to the protein molecules. However, new results show that the loss of diffractive power may instead be attributable to the production of hydrogen gas in the crystal. At 100 K it is likely that the hydrogen gathers at interfaces between crystal domains, which would account for the commonly observed increase in mosaicity with dose. However, at 50 K it has been found that the rate of the specific structural damage with dose was reduced by a factor of four, although the global damage was not much slower than at 100 K. It is thus more likely that rather than collecting at grain boundaries the hydrogen is trapped within the unit cell at 50 K, accounting for the larger unit-cell increase at this temperature (Meents *et al.*, 2010 ▶).

An important experimental consideration is that at cryotemperatures the damage does not usually spread along the crystal. The crystal can thus be translated with respect to the beam so that fresh undamaged crystal can be irradiated at multiple positions. The exception is when heavy-atom clusters are present. The high absorption of these atoms can result in local heating of the crystal above 110 K, at which diffusion of 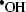 radicals becomes more probable (*e.g.* for Na^+^,K^+^-ATPase crystals soaked with Ta_6_Br_12_^2+^; Poul Nissen and J. Preben Morth, private communication at the Petra-III Workshop 2007). In this case, translation of the crystal to a new position can be unsuccessful as a strategy for obtaining more data, since the damage can spread several tens of micrometres along the crystal from the irradiated position.

With the advent of X-ray microbeams, the question arises as to how close sequential irradiations can be made while ensuring that ‘fresh’ material is in the beam, and there is ongoing systematic research to investigate this (Robert Fischetti, private communication).

## 4. Why should we care?

Radiation damage in MX is an increasingly important and limiting problem for several reasons. Firstly, as the diffraction experiment proceeds, creeping non-isomorphism occurs on three simultaneous fronts: the unit-cell volume increases, there is often movement of the protein molecule within the unit cell, and structural changes are induced by the damage, so that the protein conformation is changing during the measurements. This non-isomorphism is thought to be a major cause of unsuccessful MAD (multiple-wavelength anomalous dispersion) structure determinations, since by the time the second or third wavelength is collected, the cell and atomic structure can have changed such that the reflection intensities are significantly altered. This effect can obscure the anomalous signal required for structure solution. It has been calculated that a 0.5% change in all three dimensions of a 100 Å^3^ unit cell would change the intensity of a 3 Å reflection by 15% (Crick & Magdoff, 1956 ▶) so the MAD/SAD phasing signals would be completely destroyed. An empirical rule of thumb for successful MIR phasing has been proposed for the absolute shift in unit-cell dimensions (*X*) that can be tolerated as a function of the resolution limit of the data set (*d*_min_): *X* = *d*_min_/4 (Drenth, 1999 ▶).

Secondly, the radiation-sensitivity of some crystals at 100 K means that it is not possible to collect a complete data set from a single crystal and data must be merged from several (or many) of them to measure all the unique reflection intensities. Although this was routinely the case when data were collected at RT, most crystallographers have become accustomed to being able to measure all unique reflections from just one cryocooled crystal. Use of multiple crystals to assemble a complete data set in general increases the errors arising from non-isomorphism, thereby potentially reducing the ease of structure solution as well as increasing the mounting/dismounting time burden. Even using a robot for this operation can be slow and in fact is sometimes the most time-consuming part of the experiment. It can also present some pitfalls during processing. For instance, space group *I*4 can be indexed with the *b* axis pointing in either direction, so that when data are merged care must be taken that each section is indexed in the same convention.

Finally, the radiation-damage-induced structural changes can affect the apparent biological properties of the macromolecule under study. Enzyme mechanisms can involve redox-susceptible residues, so special care is required when interpreting structures that may have been modified by X-ray damage during the data collection. For instance, irradiation can change the oxidation state of metal ions in structural/active sites from that in their native state (Carugo & Djinovic Carugo, 2005 ▶; Yano *et al.*, 2005 ▶) and cause the decarboxylation of glutamate and aspartate residues. X-ray-induced structural changes can also be misleading in studies of intermediates (*e.g.* Takeda *et al.*, 2004 ▶). In such circumstances, separating radiation damage from an enzymatic mechanism can be extremely difficult and can cast doubt on the validity of biological conclusions drawn from crystal structures (Ravelli & Garman, 2006 ▶).

In summary, radiation damage ultimately results in lower resolution structures, failed MAD structure solutions and sometimes the inaccurate interpretation of biological results if no control experiments are carried out to account for radiation-damage artefacts. It is thus an issue to be taken seriously by the structural biologist.

## 5. What is ‘dose’ and the ‘dose limit’?

As already stated, the universal metric against which the decay indicators of a crystal are conveniently measured is the absorbed dose, which is defined as the energy absorbed per unit mass of the sample (Gy = J kg^−1^) in the irradiated volume. The fact that the amount of damage at 100 K is indeed proportional to the absorption coefficient and thus to the dose has been shown in elegant experiments by Kmetko *et al.* (2006 ▶) on lysozyme crystals soaked in a range of concentrations of various heavy-atom solutions.

The ‘dose postulate’ states that there exists a universal ‘dose limit’, which is the maximum energy/mass that a macromolecular crystalline sample can tolerate before the diffraction will fade to a given level (traditionally half) of its original intensity. A crystal might not survive until the limit is reached (*e.g.* if there were susceptible residues at crystal contacts; Murray *et al.*, 2005 ▶), but it would not be expected to survive beyond it. From observations made of the dose which generally caused biological samples at 77 K to lose half of their diffracting power (*D*_1/2_) during two-dimensional diffraction experiments in electron microscopy, Henderson (1990 ▶) estimated a ‘dose limit’ (known as the ‘Henderson limit’) for three-dimensional macromolecular X-ray crystallography of 20 MGy. This was later measured experimentally in a series of experiments on apoferritin and holoferritin crystals (see Fig. 6 ▶), the absorption coefficients of which differ by a factor of two (Owen *et al.*, 2006 ▶). The composition of the crystals was determined using proton-induced X-ray emission (PIXE; Garman & Grime, 2005 ▶) in order to obtain as accurate values as possible of, in particular, the iron content. This minimized the errors in the dose calculations. The dose limit (*D*_1/2_) was found to be 43 MGy, although the recommended maximum dose was only 30 MGy in order to avoid compromising the biological information extracted from deduced structures. This dose limit corresponded to a drop in diffraction intensity to 70% (*D*_0.7_) of the initial value (Owen *et al.*, 2006 ▶). A number of other studies have corroborated this dose limit. An analysis of all the various experimental measurements has been made by Howells *et al.* (2009 ▶), who concluded that the resolution-dependent *D*_1/2_ was 10*d* MGy (where *d* is the resolution in Å: thus for a 2 Å reflection *D*_1/2_ = 20 MGy). This issue is described in detail later in this volume (Holton & Frankel, 2010 ▶). As noted above, this limit is thought to be largely independent of dose rate at cryotemperatures at the flux densities currently used in MX. It is also worth reiterating that structural damage generally occurs well before visible degradation of the diffraction pattern is observed. Thus, it is in­advisable to plan an experiment which requires collecting data beyond the time when the dose limit (which was determined from intensity decay) is reached.

In the RT model developed by Blake & Phillips (1962 ▶), damage is directly proportional to dose and no dose-rate effect is included. Despite anecdotal reports from the early days of synchrotron use with crystals irradiated at RT that they had longer lifetimes at higher dose rates, this was only systematically investigated recently, when an inverse dose-rate effect was measured in-house at RT between dose rates of 6 and 10 Gy s^−1^, the higher rate giving four times the dose tolerance (*i.e.* four times the dose required to halve the total diffraction intensity, *D*_1/2_) for hen egg-white lysozyme crystals (Southworth-Davies *et al.*, 2007 ▶). For irradiation at a dose rate of 2800 Gy s^−1^ at a synchrotron at RT, ten times the dose tolerance has been recorded (Barker *et al.*, 2009 ▶). The explanation of this phenomenon is that at high dose rates radicals produced in the crystal neutralize one another and thus do not cause further damage, whereas at lower dose rates they travel further, interacting with protein and solvent to produce additional damage.

Interestingly, the RT exponential intensity decay with dose, which is typical of a first-order process (where the decay rate depends on the amount of material left), can be modified by the addition of scavenger molecules to become a zeroth-order dependence (where the rate of decay is a constant). This effect is not yet completely understood. At RT, the dose tolerance of HEWL crystals (as measured by the change in *D*_1/2_) has been shown to be improved by factors of ∼2 and ∼9 by the addition of the scavengers ascorbate and 1,4-benzoquinone, respectively (Barker *et al.*, 2009 ▶).

To calculate the available time in the beam before the crystal reaches the experimental dose limit, knowledge of the sample size and composition (*i.e.* the number of each atom type in the unit cell) is required so that absorption coefficients can be computed, as well as detailed information about the incident beam [energy, size, shape and flux (in photons s^−1^)]. For MX, this can be conveniently carried out by means of the program *RADDOSE* (Murray *et al.*, 2004 ▶; Paithankar *et al.*, 2009 ▶; Paithankar & Garman, 2010 ▶), version 3 of which includes both the probability of fluorescent X-ray escape (non-negligible for heavy-atom-containing crystals) and the energy loss owing to Compton scattering (non-negligible above 20 keV). The calculations rely on accurate flux measurements being available for the X-ray beam at the particular beamline being used (Owen, Holton *et al.*, 2009 ▶). However, *RADDOSE* does not yet give accurate results for crystals larger than the beam size where a fresh unirradiated crystal is continually being rotated into the beam. The time before the experimental limit is reached is thus underestimated in these cases. Currently, developments are under way that aim to provide on-line digitization of both the crystal shape and its position relative to the rotation axis of the goniometer. These efforts are being largely driven by the need for improved absorption corrections, but when the crystal information can be incorporated into *RADDOSE* they should also make possible the further improvement of dose estimates.

## 6. What do we know and what would we like to know?

There are many parameters that can be varied in an MX experiment, some of which can affect the rate of radiation damage to (or ‘dose tolerance’ of) a crystal. There are two challenges for researchers seeking to understand and trying to mitigate radiation damage. The first is to truly isolate the variable to be tested and to only change one experimental condition at a time so that definite conclusions can be reached. The second is to use a reliable metric(s) of radiation damage so that the effect of protocol modifications can be properly assessed. In addition, to reach a statistically significant result the same experiments must be repeated and reproduced on several crystals of the same protein and ideally extended to check the validity of the results in a more general way by conducting the same tests for a number of different proteins.

Over the last 10 years there has been an extensive search for reliable metrics of global and structurally specific radiation damage. Since structural changes occur even before degradation of diffraction quality is apparent, intensity loss cannot be used as a yardstick with which to judge damage to specific amino acids, which is only obvious when the electron-density maps have been calculated once enough data have been collected. This can be understood because the diffraction loss occurs in reciprocal space and the specific damage in real space, and one point in real space contributes to all reflections in reciprocal space and *vice versa*.

The parameter space of an MX experiment is composed of variables that can be categorized as follows (Garman, 2003 ▶).

(i) The crystal in the cryo-loop: heavy-atom content (Se, S *etc.*), solvent content, solvent composition, crystal size and surface-to-volume ratio, the amount of residual liquid around the crystal prior to flash-cooling, the choice and concentration of cryoprotectant agent, the time spent in the cryobuffer prior to cooling, the flash-cooling method (stream or liquid), the cryogen used to flash-cool, the amount of crystal manipulation, the local humidity and the speed of the experimenter when flash-cooling from the cryobuffer drop.
(ii) The X-ray beam: the flux density, the energy (wavelength), the beam size compared with the crystal size, the dose and the dose rate.
(iii) The cryostat: the cold gas flow rate, the temperature and the cryogen (N_2_ or He).

Systematic experiments to address the dependence of the rate of radiation damage on all these factors would take many years and be very labour-intensive in terms of data collection and processing, as well as requiring many hours of synchrotron beamtime. However, some of these variables have been investigated and studies can be broadly categorized as follows.

(i) Crystal related.
  What is the minimum crystal size?
  What affects X-ray absorption?
  Can the unit-cell expansion be used as a metric?
  What is the effect of temperature (*e.g.* 100, 16, 40 K)?
  Does the addition of free-radical scavengers increase dose tolerance?
  What are the susceptibilities of particular amino acids to specific damage and why?
(ii) X-ray beam related.
  What is the effect of changing the incident wavelength?
  Is it beneficial to change/regulate the dose/dose-rate regime?
  What is the effect of the beam size compared with the crystal size?
  Does the beam heat the crystal?
(iii) Method developments and applications.
  Development of convenient flux calibration of beamlines.
  Development of on-line and off-line spectroscopy (UV–vis, Raman, XAS, EPR).
  Studying the effect on the success of MAD/SAD phasing.
  Development of RIP/RIPAS.
  Application/effect of radiation damage to/on the study of biological mechanisms.
  Phase transitions and/or radiation-induced changes with temperature-controlled cryocrystallography to study macromolecular function.
  Software developments.
  Finding strategies to minimize radiation damage in data collections.
  Extending the understanding of radiation damage in RT data collections.
  Studying RNA/DNA damage.

The many experiments on the above topics that have been reported to date will not be detailed here, but a summary of the currently available literature is presented in Table 1 ▶. Useful collections of research papers addressing different aspects of radiation damage in MX can be found in four special issues of the *Journal of Synchrotron Radiation*, which each contain eight or more research papers presented at the Second, Third, Fourth and Fifth International Workshops on X-ray Radiation Damage to Biological Crystalline Samples [*Journal of Synchrotron Radiation*, Vol. **9** (2002), pp. 327–381, Vol. **12** (2005 ▶), pp. 257–328, Vol. **14** (2007), pp. 1–132 and Vol. **16** (2009), pp. 129–216, respectively]. These each have a brief introduction placing the contributions into the wider context of research in the field (Garman & Nave, 2002 ▶, 2009 ▶; Nave & Garman, 2005 ▶; Garman & McSweeney, 2007 ▶).

The real question to which experimenters would like an answer is: what can I do to obtain the largest amount of data with the highest signal-to-noise ratio from my crystal in the beam? The current advice would include the following: (i) backsoaking of crystals to remove any nonspecifically bound heavy atoms in the mother liquor (*e.g.* the arsenic in cacodylate) or in a soaking solution for heavy-atom phasing, since these heavy atoms can contribute a lot to the dose owing to their high absorption but do not provide useful phasing information; (ii) sacrificing a crystal (if more than one crystal of a protein exists) to obtain a data set where the aim is to assess the radiation-sensitivity so that a suitable data-collection protocol can be designed; (iii) matching the beam size to the crystal size; (iv) if possible using a beam with a top-hat profile (or by careful slit setting selecting the central peak portion of a Gaussian-shaped beam) so that the crystal does not suffer differential radiation damage across its irradiated volume; (v) using *BEST* to optimize the data-collection strategy taking radiation damage into account (Bourenkov & Popov, 2010 ▶) and (vi) being satisfied with a 3 Å comparatively undamaged data set for phasing rather than chasing the 2.5 Å diffraction which fades as you watch and will thus be less useful.

Above all, experimenters should make themselves aware of the parameters known to affect the rate of radiation damage, so that intelligent choices/compromises can be made.

## 7. Conclusions

Since systematic research into MX radiation damage at 100 K began in earnest in the late 1990s, significant progress has been made in our knowledge and understanding of the phenomenon and much anecdotal evidence has been replaced by solid experimental results. We understand better how to perform investigations to identify suitable metrics and the importance of routinely measuring the X-ray flux so that the absorbed dose can be calculated. The research has also prompted some exciting new approaches such as RIP, UV-RIP/RIPAS, ‘time-resolved’ cryocrystallography and on-line spectroscopy. However, there are still many areas where systematic investigations are required to improve our understanding of the radiation chemistry within an irradiated protein crystal held at either RT or at various cryotemperatures so that better strategies for minimizing damage can be developed.

The most useful contribution to be made by MX radiation-damage research is in identifying concrete experimental protocols for everyday use on synchrotron beamlines so that researchers can ensure that they obtain the maximum possible high-quality data from their crystals. This would firstly facilitate structure solution and secondly avoid compromising the biological information extracted from the structure once obtained.

## Figures and Tables

**Table 1:** Compendium of the current literature on MX radiation damage Every effort has been made to make this compilation exhaustive: a copy is maintained and updated at the URL http://www.biop.ox.ac.uk/www/garman/gindex.html. References to the pre-1990 papers on RT MX damage studies can be found in §[Sec sec1]1.

Crystal-related questions	
What is the minimum crystal size?	Gonzalez & Nave (1994 ▶), Teng & Moffat (2000 ▶), Glaeser *et al.* (2000 ▶), Sliz *et al.* (2003 ▶), Nave & Hill (2005 ▶), Cowan & Nave (2008 ▶), Moukhametzianov *et al.* (2008 ▶), Holton (2009 ▶), Holton & Frankel (2010 ▶)
What affects X-ray absorption? (and *RADDOSE*)	Murray *et al.* (2004 ▶, 2005 ▶), Kmetko *et al.* (2006 ▶), Holton (2007 ▶, 2009 ▶), Paithankar *et al.* (2009 ▶), Paithankar & Garman (2010 ▶)
Can the unit-cell expansion be used as a metric?	Teng & Moffat (2000 ▶), Murray & Garman (2002 ▶), Ravelli *et al.* (2002 ▶), Müller *et al.* (2002 ▶)
What is the effect of temperature (*e.g.* 100, 16, 40 K)?	Garman (1999 ▶), Hanson *et al.* (1999 ▶, 2002 ▶), Weik *et al.* (2001 ▶), Teng & Moffat (2002 ▶), Yano *et al.* (2005 ▶), Grablolle *et al.* (2006 ▶), Borek *et al.* (2007 ▶), Meents *et al.* (2007 ▶, 2010 ▶), Chinte *et al.* (2007 ▶), Corbett *et al.* (2007 ▶), Colletier *et al.* (2008 ▶)
Does the addition of radical scavengers increase dose tolerance?	Murray & Garman (2002 ▶), Betts (2004 ▶), Kauffmann *et al.* (2006 ▶), Borek *et al.* (2007 ▶), Southworth-Davies & Garman (2007 ▶), Holton (2007 ▶), Macedo *et al.* (2009 ▶), Barker *et al.* (2009 ▶), Nowak *et al.* (2009 ▶)
What are the susceptibilities of particular amino acids to specific damage and why?	Weik *et al.* (2000 ▶), Ravelli & McSweeney (2000 ▶), Burmeister (2000 ▶), Leiros *et al.* (2001 ▶), Fioravanti *et al.* (2007 ▶)

X-ray beam-related questions	
What is the effect of changing the incident wavelength?	Arndt (1984 ▶), Gonzalez & Nave (1994 ▶), Murray *et al.* (2004 ▶), Weiss *et al.* (2005 ▶), Shimizu *et al.* (2007 ▶)
Is it beneficial to change/regulate the dose/dose-rate regime?	Teng & Moffat (2000 ▶), O’Neill *et al.* (2002 ▶), Sliz *et al.* (2003 ▶), Ravelli *et al.* (2002 ▶), Leiros *et al.* (2006 ▶), Owen *et al.* (2006 ▶), Howells *et al.* (2009 ▶)
What is the effect of the beam size compared with the crystal size?	Schulze-Briese *et al.* (2005 ▶)
Does the beam heat the crystal?	Kuzay *et al.* (2001 ▶), Nicholson *et al.* (2001 ▶), Müller *et al.* (2002 ▶), Snell *et al.* (2002 ▶, 2005 ▶, 2007 ▶), Kriminski *et al.* (2003 ▶), Mhaisekar *et al.* (2005 ▶)

Methods developments and applications	
Development of convenient flux calibration of beamlines	Owen *et al.* (2009 ▶)
Development of on-line and off-line spectroscopy (UV–vis, Raman, XAS, EPR)	Weik *et al.* (2002 ▶), Murray & Garman (2002 ▶), Matsui *et al.* (2002 ▶), Takeda *et al.* (2004 ▶), Sato *et al.* (2004 ▶), Adam *et al.* (2004 ▶, 2009 ▶), Yano *et al.* (2005 ▶), Dubnovitsky *et al.* (2005 ▶), Carpentier *et al.* (2007 ▶), McGeehan *et al.* (2007 ▶, 2009 ▶), Pearson *et al.* (2007 ▶), Corbett *et al.* (2007 ▶), Holton (2007 ▶), Hough *et al.* (2008 ▶), Utschig *et al.* (2008 ▶), Owen *et al.* (2009 ▶)
Studying the effect on the success of MAD/SAD phasing	Rice *et al.* (2000 ▶), Schiltz *et al.* (2004 ▶), Zwart *et al.* (2004 ▶), González *et al.* (2005 ▶), González (2007 ▶), Ramagopal *et al.* (2005 ▶), Ravelli *et al.* (2005 ▶), Oliéric *et al.* (2007 ▶)
Development of RIP/RIPAS	Ravelli *et al.* (2003 ▶, 2005 ▶), Banumathi *et al.* (2004 ▶), Weiss *et al.* (2004 ▶), Nanao *et al.* (2005 ▶), Nanao & Ravelli (2006 ▶), Schiltz & Bricogne (2007 ▶), Rudiño-Piñera *et al.* (2007 ▶), Fütterer *et al.* (2008 ▶), Schönfeld *et al.* (2008 ▶)
Application/effect of radiation damage to/on the study of biological mechanisms	Matsui *et al.* (2002 ▶), Alphey *et al.* (2003 ▶), Nukaga *et al.* (2003 ▶), Mees *et al.* (2004 ▶), Takeda *et al.* (2004 ▶), Kort *et al.* (2004 ▶), Roberts *et al.* (2005 ▶), Dubnovitsky *et al.* (2005 ▶), Sjöblom *et al.* (2009 ▶), Adam *et al.* (2009 ▶)
Metalloproteins	Schlichting *et al.* (2000 ▶), Berglund *et al.* (2002 ▶), Adam *et al.* (2004 ▶), Baxter *et al.* (2004 ▶), Sato *et al.* (2004 ▶), Carugo & Djinovic Carugo (2005 ▶), Yano *et al.* (2005 ▶), Echalier *et al.* (2006 ▶), Pearson *et al.* (2007 ▶), Beitlich *et al.* (2007 ▶), Kühnel *et al.* (2007 ▶), Corbett *et al.* (2007 ▶), Hough *et al.* (2008 ▶), Petrova *et al.* (2009 ▶)
Phase transitions and/or radiation-induced changes with temperature-controlled cryocrystallography to study macromolecular function	Schlichting *et al.* (2000 ▶), Weik, Kryger *et al.* (2001 ▶), Weik, Ravelli *et al.* (2001 ▶), Weik *et al.* (2005 ▶), Hersleth *et al.* (2008 ▶), Colletier *et al.* (2008 ▶)
Software developments	Diederichs *et al.* (2003 ▶), Nanao *et al.* (2005 ▶), Bourenkov & Popov (2006 ▶, 2010 ▶), Diederichs (2006 ▶), Schiltz & Bricogne (2007 ▶)
Finding strategies to minimize radiation damage in data collections	Berglund *et al.* (2002 ▶), Adam *et al.* (2004 ▶), Stern *et al.* (2009 ▶), Incardona *et al.* (2009 ▶), Borek *et al.* (2010 ▶)
Extending the understanding of radiation damage in RT data collections	Southworth-Davies *et al.* (2007 ▶), Barker *et al.* (2009 ▶)
Studying RNA/DNA damage	Ennifar *et al.* (2002 ▶), Mees *et al.* (2004 ▶), Schiltz *et al.* (2004 ▶), Oliéric *et al.* (2007 ▶), McGeehan *et al.* (2007 ▶)
